# Beyond Surgical Technique: Precision, Personalization and Quality of Care in Oral and Maxillofacial Surgery

**DOI:** 10.3390/medicina62071295

**Published:** 2026-07-05

**Authors:** Luca Aquilanti, Giorgio Rappelli

**Affiliations:** 1 Department of Clinical Specialistic and Dental Sciences, Polytechnic University of Marche, Via Tronto, 10/A, 60126 Ancona, Italy; 2 Dentistry Clinic, National Institute of Health and Science of Ageing, IRCCS INRCA, Via Tronto, 10/A, 60126 Ancona, Italy

Oral and maxillofacial surgery has undergone significant changes over the past decade. Technological innovation, advances in regenerative medicine, improved diagnostic imaging, and an increasing focus on patient-centered care have broadened the role of the oral and maxillofacial surgeon beyond technical execution. Contemporary surgical practice increasingly relies on accurate diagnosis, individualized treatment planning, multidisciplinary collaboration, and evidence-based decision-making to achieve predictable clinical outcomes while minimizing patient morbidity.

At the same time, the scope of the specialty has broadened considerably. The refinement of established surgical procedures has been accompanied by new technologies and treatment strategies, expanding therapeutic possibilities while creating new challenges in diagnosis, preoperative planning, and perioperative management. Consequently, successful treatment is no longer determined solely by surgical expertise but also by the ability to integrate clinical information, imaging findings, biological principles, and patient-related factors into a comprehensive treatment strategy.

The studies in this Special Issue reflect this evolution from different perspectives. Although they address heterogeneous clinical conditions and research questions, together they highlight several themes that currently characterize oral and maxillofacial surgery, including surgical innovation, optimization of surgical planning, individualized perioperative management, emerging clinical challenges, and quality of care. Rather than focusing on a single procedure or subspecialty, the published studies emphasize the multifaceted nature of contemporary surgical practice, where treatment outcomes increasingly depend on the integration of accurate diagnosis, appropriate patient selection, evidence-based protocols, and careful postoperative management.

This Editorial discusses the main themes emerging from the Special Issue within the broader context of the ongoing evolution of oral and maxillofacial surgery. Although the published studies investigate different aspects of clinical practice, they consistently suggest that achieving optimal surgical outcomes increasingly depends on factors extending well beyond the technical aspects of the surgical procedure itself, including treatment planning, individualized decision-making, and the overall quality of care.

One of the defining characteristics of current oral and maxillofacial surgery is the progressive shift in surgical decision-making toward the preoperative phase. Surgical precision increasingly depends not only on manual skills and intraoperative experience but also on the quality of diagnosis, the accuracy of preoperative planning, and the integration of digital technologies into clinical workflows. Consequently, the surgical procedure has become one component of a broader process that begins well before the patient enters the operating room.

This concept is exemplified by several studies published in this Special Issue. Digital planning and point-of-care manufacturing were successfully integrated into the rehabilitation of oral cancer patients through patient-specific surgical guides, demonstrating how hospital-based production workflows can improve the efficiency and flexibility of implant rehabilitation in complex clinical scenarios [1]. Likewise, the Titanium Alveolar Customized Osteogenic Scaffold (TACOS) protocol highlights the importance of a standardized workflow for vertical ridge augmentation, emphasizing that predictable regenerative outcomes depend not only on customized devices but also on the meticulous execution of every surgical step, from graft preparation to soft tissue management [2].

The study evaluating the three-dimensional relationship between impacted mandibular third molars and the inferior alveolar canal further reinforces the importance of preoperative planning [3]. Detailed CBCT assessment contributes to more informed clinical decision-making and improved risk assessment before surgery.

Rather than changing the objectives of surgery, these innovations make surgical procedures more predictable, reproducible, and tailored to the individual clinical scenario. Together, these studies demonstrate that precision in oral and maxillofacial surgery increasingly derives from the integration of accurate diagnosis, structured planning, and standardized clinical workflows rather than from technical execution alone.

The growing importance of planning has been accompanied by an equally significant evolution in patient management. Current clinical practice increasingly recognizes that successful treatment cannot be defined solely by technical outcomes. Instead, the selection of surgical techniques, biomaterials, and perioperative protocols should reflect each patient’s clinical characteristics and risk profile to reduce complications while preserving treatment effectiveness.

This concept is reflected in the study investigating postoperative bleeding in patients receiving oral anticoagulant therapy [4]. Rather than comparing anticoagulant regimens, the authors identified patient- and treatment-related factors associated with postoperative bleeding, highlighting the importance of individualized risk assessment in everyday clinical practice. This approach encourages clinicians to consider perioperative risk as an integral component of treatment planning rather than as an isolated intraoperative concern.

A similar perspective emerges from the scoping review on cyanoacrylate tissue adhesives in oral surgery and periodontology [5]. The review suggests that adjunctive surgical strategies should be selected according to procedure-specific characteristics and expected clinical benefits rather than routine preferences. Likewise, the systematic review comparing piezoelectric surgery with conventional rotary instruments for mandibular third molar removal emphasizes that innovative surgical technologies should be adopted because of their ability to improve patient-centered outcomes, including postoperative pain, swelling, and overall morbidity, rather than for technological novelty alone [6].

Taken together, these contributions reflect a broader transition from standardized surgical protocols to individualized care. Modern oral and maxillofacial surgery increasingly requires treatment strategies that integrate scientific evidence, patient-related factors, and clinical judgment to optimize outcomes while minimizing unnecessary morbidity. Ultimately, the value of new techniques and materials lies not simply in their availability but in their appropriate application according to the needs of each individual patient.

While innovation and individualized treatment have become central components of modern surgical practice, oral and maxillofacial surgery is also continuously influenced by broader clinical and societal factors. Changes in healthcare systems, the emergence of new disease entities, and the epidemiology of maxillofacial conditions all contribute to shaping surgical decision-making and, ultimately, patient outcomes.

A broader perspective emerges from the retrospective analysis evaluating the impact of the COVID-19 pandemic on patients undergoing surgery for oral squamous cell carcinoma [7]. The study illustrates how disruptions in healthcare delivery may influence disease presentation, leading to more advanced tumors at diagnosis and consequently more complex surgical management. These findings emphasize that treatment outcomes depend not only on surgical expertise but also on timely diagnosis, appropriate referral pathways, and equitable access to specialized care.

The narrative review on post-COVID-19 jaw osteonecrosis (PC-ONJ) further highlights the dynamic nature of the specialty, emphasizing the importance of maintaining a critical and adaptive approach when new clinical challenges arise [8]. As the understanding of newly recognized diseases evolves, clinicians are increasingly required to integrate emerging evidence with established diagnostic and therapeutic principles.

A complementary perspective is provided by the epidemiological study on orbital wall disorders [9]. Although epidemiological investigations do not directly evaluate surgical techniques, they provide valuable information for identifying population needs, allocating healthcare resources, and developing preventive strategies. Understanding disease distribution and injury patterns remains an essential component of healthcare planning and reinforces the broader role of oral and maxillofacial surgery within contemporary healthcare systems.

Taken together, these studies remind us that advances in oral and maxillofacial surgery cannot be measured solely by the introduction of new techniques. Equally important is the ability to respond to changing clinical scenarios while maintaining high standards of patient care.

The central message emerging from these contributions is that technical excellence alone no longer defines quality in oral and maxillofacial surgery. Accurate diagnosis, careful planning, individualized patient management, and the ability to adapt to evolving clinical scenarios have become equally important components of successful surgical care. Advances in technology and biomaterials provide new opportunities, but their clinical value ultimately depends on thoughtful integration into evidence-based practice.

Future research should focus not only on developing new surgical techniques but also on improving clinical decision-making, strengthening multidisciplinary collaboration, and generating robust evidence capable of supporting personalized treatment strategies. Innovation should ultimately serve to improve patient care rather than become an objective in itself.

Rather than providing definitive answers, the contributions collected in this Special Issue encourage continued reflection on current clinical practice and underscore the importance of questioning, refining, and improving existing approaches. This commitment to critical thinking and continuous improvement has always driven progress in oral and maxillofacial surgery and will remain essential for meeting the challenges of the years ahead.
